# Prevalence of Anterior Inferior Cerebellar Artery Vascular Loop in Cerebellopontine Angle With Three-Dimensional Constructive Interference in Steady State (CISS) Sequence MRI

**DOI:** 10.7759/cureus.61393

**Published:** 2024-05-30

**Authors:** Nithish G, Samanvitha H, Shantkumar S Sajjan, Harsha M T, Monika S

**Affiliations:** 1 Department of Diagnostic Radiology, Bangalore Medical College and Research Institute, Bengaluru, IND; 2 Department of Interventional Radiology, All India Institute of Medical Sciences, Rishikesh, Rishikesh, IND; 3 Department of Diagnostic and Interventional Radiology, Postgraduate Institute of Medical Education and Research, Chandigarh, IND; 4 Department of Ear, Nose, and Throat (ENT) & Head and Neck Surgery (HNS), Bangalore Medical College and Research Institute, Bengaluru, IND

**Keywords:** 3d-ciss mri, asymptomatic, vascular loop, internal auditory canal, cerebellopontine angle cistern, anterior inferior cerebellar artery

## Abstract

Background

The cerebellopontine angle (CPA) cistern houses vital neurovascular structures such as cranial nerves V, VII, and VIII and the anterior inferior cerebellar artery (AICA), often leading to neurovascular compression syndromes due to its complex anatomy. Although vascular compression is a recognized cause of certain neuralgias, its association with otologic symptoms such as tinnitus, hearing loss, and dizziness remains uncertain. Hence, this study aims to determine the prevalence of the AICA vascular loop in the CPA cistern on MRI in patients with asymptomatic audiovestibular symptoms.

Methodology

Adult patients who underwent MRI, including the posterior fossa’s high-resolution volumetric T2 sequence (three-dimensional constructive interference in steady state (3D-CISS)), were assessed. Patients with a history of audiovestibular symptoms (tinnitus/dizziness/vertigo/sensorineural hearing loss), intracranial tumor, vascular lesions, intracranial surgery, brain radiation therapy, traumatic brain injury, poor image quality, and MRI scans without 3D-CISS sequences were excluded. Two radiologists independently reviewed 114 (228 sides) MRI studies for the vascular loop of AICA in the CPA cistern and the extension of the AICA loop into the ipsilateral internal acoustic meatus which was graded by Chavda’s classification.

Results

The prevalence of vascular loop of AICA in the CPA cistern was as high as 47.6% in asymptomatic patients. Grade I Chavda vascular loop was the most common type followed by type II, with type III being the least common type.

Conclusions

Knowledge regarding the high prevalence of the AICA loop in the asymptomatic population and the lack of significant correlation between the presence of the AICA loop and otovestibular symptoms should be considered in preoperative planning for decompression procedures.

## Introduction

The cerebellopontine angle (CPA) cistern is a paired basal cistern at the CPA in which the vascular and neural structures highly interact. The neurovascular structures in CPA include cranial nerves V, VII, and VIII; anterior inferior cerebellar artery (AICA); auditory artery; branches of the petrosal vein; vein of the middle cerebellar peduncle; vein of the lateral recess of the fourth ventricle; and transverse pontine vein [1]. Anatomical interactions between these elements may result in neurovascular compression syndromes.

AICA usually originates from the basilar artery about 1 cm above the junction of the two vertebral arteries and courses laterally, crossing the cranial nerve VIII within the CPA cistern [2]. A loop of AICA also enters the internal auditory canal (IAC) along with VII and VIII cranial nerves in about 20-40% of cases. This anatomical course of AICA makes it the culprit vessel implicated in various compression syndromes [3]. The term “vascular compression syndrome” was first described by McKenzie in 1936 and made popular by Jannetta in 1975. It refers to a group of diseases caused by direct contact between a blood vessel and a cranial nerve [4].

Although vascular contacts at the root exit zone of the trigeminal, facial, and glossopharyngeal nerves have been widely accepted as a cause of trigeminal neuralgia, hemifacial spasm (HFS), and glossopharyngeal neuralgia, respectively, their relationship to otologic symptoms such as tinnitus, hearing loss, and dizziness remains unclear [5]. The prevalence of otological symptoms is 2% for tinnitus, 66% for sensorineural hearing loss, and 11% for dizziness in the South Indian population [1]. While various diseases are linked to otologic symptoms, the exact etiology is not always known [6]. MRI is considered the modality of choice for unexplained otological symptoms and is used to exclude retro-cochlear pathologies, especially in patients with asymmetrical sensorineural hearing loss, unilateral tinnitus, or vestibular findings [7].

The World Health Organization estimates that by 2050, hearing loss will approach approximately 2.5 billion, with 700 million people requiring intervention [8]. Although various variables, including infections, vascular, immunological, metabolic, neoplastic, ototoxic, and traumatic events, have been linked to cochlear damage, precise etiology is typically unknown [5].

There is insufficient information to address the frequency with which a vessel is contacting or compressing the vestibulocochlear nerve (cranial nerve VIII) as an incidental finding in the absence of any otological history, despite investigators having studied the accuracy of MRI for identifying vascular loop compression preoperatively compared with the gold standard of intraoperative findings during microvascular decompression for HFS. Vascular loops have long been attributed as a potential cause of unexplained tinnitus/idiopathic sensorineural hearing loss. Few studies have tried to find a correlation between otological symptoms and attribute these to the vascular loops in the CPA cistern/IAC. However, no study has shown the prevalence of such vascular loops contacting/compressing the VII and VIII nerve complex as an incidental finding in asymptomatic patients. Hence, this study aims to determine the prevalence of vascular loops of AICA in the CPA cistern and IAC on MRI in patients asymptomatic for audiovestibular symptoms.

## Materials and methods

**Figure 1:**
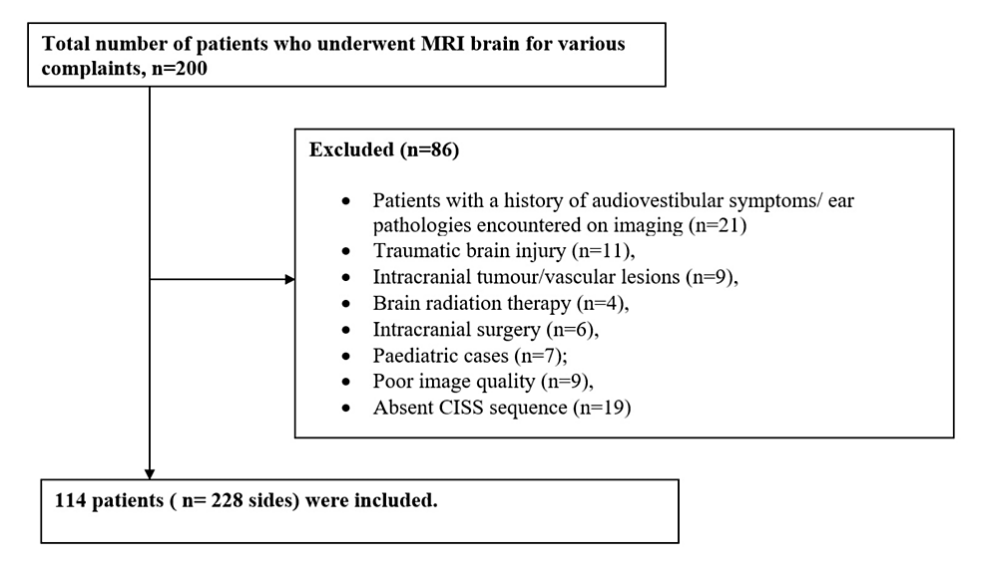
Flowchart of the study population. CISS = constructive interference in steady state

A retrospective study was conducted by accessing the radiology database and the need for informed consent was waived. MRI studies of adult patients (age >18 years) that included high-resolution volumetric T2 sequence, that is, three-dimensional-constructive interference in steady state (3D-CISS) sequence of the posterior fossa were assessed. Patients with audiovestibular symptoms (tinnitus/dizziness/vertigo/sensorineural hearing loss), a history of specific conditions (intracranial tumor, vascular lesions, intracranial surgery, brain radiation therapy, traumatic brain injury), or poor image quality and MRI scans without 3D- CISS sequences were excluded. The predominant indications for the MRI scan were headache, seizures, and visual symptoms. A total of 200 consecutive MRI brain studies of all patients were evaluated. Patients with a history of audiovestibular symptoms/ear pathologies encountered on imaging (n = 21), traumatic brain injury (n = 11), intracranial tumor/vascular lesions (n = 9), brain radiation therapy (n = 4), intracranial surgery (n = 6), pediatric cases (n = 7), poor image quality (n = 9), and absent CISS sequence (n = 19) were excluded. A total of 114 patients (228 sides) who met the eligibility criteria were independently reviewed by two radiologists (Figure 1).

The volumetric high-resolution MRI sequence (3D-CISS) of the posterior fossa was acquired with one of the following two MRI scanners: (1) 1.5 Tesla Seimens Magnetom Avanto MR system (Siemens AG, Berlin/Munich, Germany) - axial CISS sequence (images through the IACs and brainstem: repetition time (TR) = 1,200 ms, time to echo (TE) = 268 ms, flip angle = 150 degrees, matrix = 320 × 256, field of view (FOV) = 240 mm, no saturation, 0.7 mm axial slice thickness with coronal and sagittal reformatted images); (2) 1.5 Tesla United Imaging uMR-570LH MR system (United Imaging, Houston, Texas, USA) - T2 axial SPAIR_CISS sequence (spectral attenuated inversion recovery constructive interference in steady state images through the internal auditory canals and brainstem: TR = 1,300 ms, TE = 240 ms, flip angle = 150 degrees, matrix = 352 × 317, FOV = 180 × 200 mm, no saturation, 0.68 mm axial slice thickness with sagittal and coronal reformatted images).

**Figure 2:**
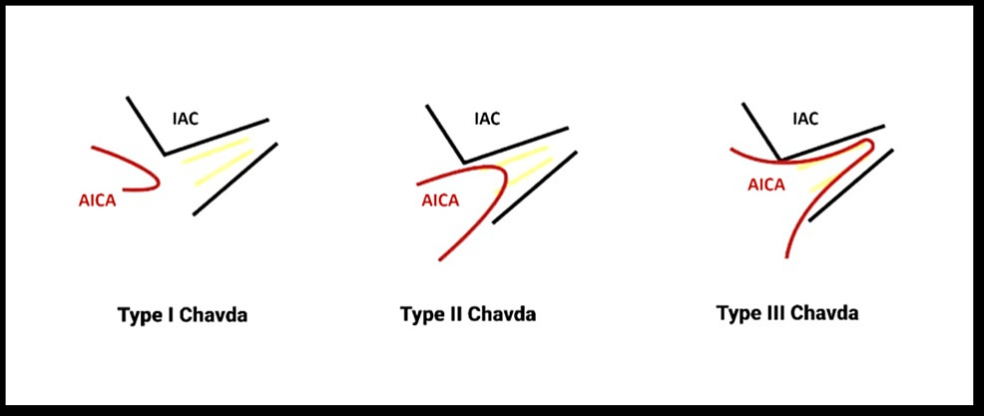
Chavda classification of the AICA vascular loop in the CPA cistern and IAC. This figure is the authors’ creation. However, the idea for the image has been taken from the author Aqeel Alameer and the website https://radiopaedia.org/cases/aica-loop-classification. CPA = cerebellopontine angle; AICA = anterior inferior cerebellar artery; IAC = internal auditory canal

3D-CISS sequence greatly improves the contrast between the cerebrospinal fluid, cranial nerves, and vessels in the CPA cistern. The reviewing radiologists were aware of the study objectives but blinded to the clinical history of audiovestibular symptoms. 3D-CISS MRI pulse sequence and multiplanar reconstructions were assessed for the vascular loop of AICA in the CPA cistern and the extension of the AICA loop into the ipsilateral internal acoustic meatus. The AICA loop was graded according to the Chavda classification [9] (Figure 2, Table 1).

**Table 1:** Chavda classification of the AICA vascular loop in the CPA cistern and IAC. AICA = anterior inferior cerebellar artery; CPA = cerebellopontine angle; IAC = internal auditory canal

Type	Description
Type I	When the AICA loop is lying within CPA but not entering the IAC
Type II	When the AICA loop enters the IAC but does not extend >50% of the length of the IAC
Type III	When the AICA loop extends >50% of the IAC

Patient demographics were described using descriptive statistics. The Kolmogorov-Smirnov test was used to check the normal distribution of the data. Continuous variables were expressed as mean, standard deviation (SD), median, and interquartile range (IQR). Ordinal or categorical variables were expressed as frequency (n) and percentage (%). The kappa value was used to assess interobserver variation/agreement levels. The kappa result was interpreted as follows: values ≤0 indicating no agreement, 0.01-0.20 indicating none to slight, 0.21-0.40 indicating fair, 0.41-0.60 indicating moderate, 0.61-0.80 indicating substantial, and 0.81-1.00 indicating almost perfect agreement [10]. A p-value <0.05 was considered statistically significant.

## Results

The mean age of the participants was 42.5 years (range = 18-81 years). There were 43 (37.7%) men and 71 (62.3%) women. Results of the assessment of the vascular loop of AICA in CPA and IAC are reported in Table 2.

**Table 2:** Results of the assessment of the vascular loop of AICA in CPA and IAC. The data are represented as frequency (n), percentage (%), and mean. Sample size of the study, n = 228. AICA = anterior inferior cerebellar artery; CPA = cerebellopontine angle; IAC = internal auditory canal

Variable		Radiologist 1, n (%)	Radiologist 2, n (%)	Mean, n (%)
Presence of the AICA loop	No	120 (52.6%)	119 (52.2%)	119.5 (52.4%)
	Yes	108 (47.4%)	109 (47.8%)	108.5 (47.6%)
Chavda classification of the AICA loop	Type 1	59 (54.6%)	62 (56.9%)	60.5 (55.8%)
	Type 2	45 (41.7%)	45 (41.3%)	45 (41.5%)
	Type 3	4 (3.7%)	2 (1.8%)	3 (2.7%)

**Figure 3:**
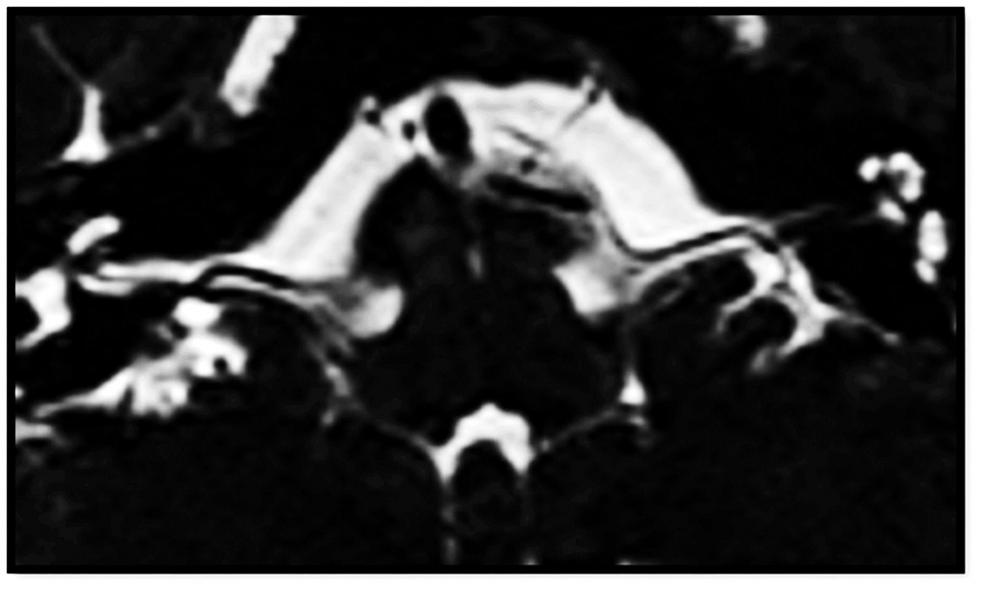
Chavda type I vascular loop. Axial high-resolution T2-weighted 3D-CISS sequence demonstrates bilateral type I Chavda vascular loop in the CPA cisterns. AICA vascular loop borders the internal auditory meatus without entering the IAC. CISS = constructive interference in steady state; CPA = cerebellopontine angle; AICA = anterior inferior cerebellar artery; IAC = internal auditory canal

**Figure 4:**
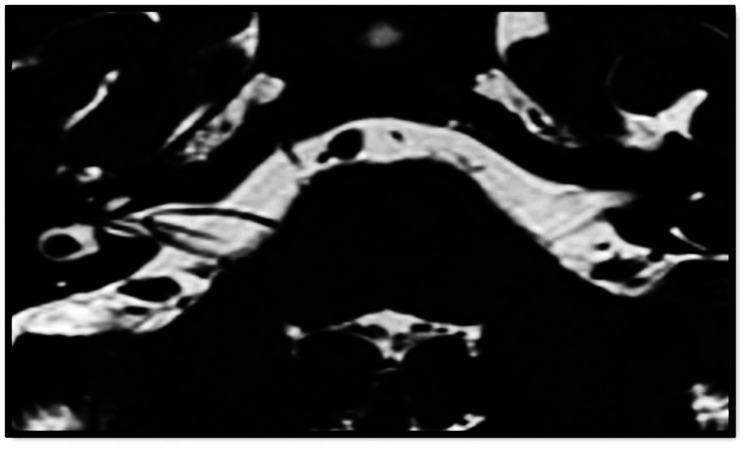
Chavda type II vascular loop. Axial high-resolution T2-weighted 3D-CISS sequence demonstrates the right AICA loop extending into the IAC (<50%). CISS = constructive interference in steady state; AICA = anterior inferior cerebellar artery; IAC = internal auditory canal

**Figure 5:**
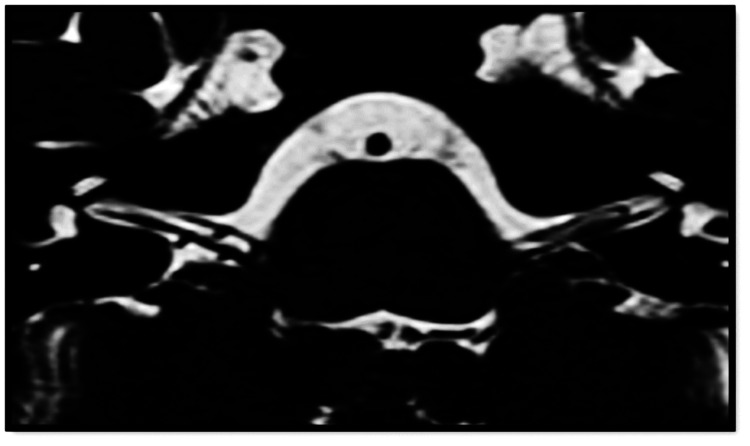
Chavda type III vascular loop. Axial high-resolution T2-weighted 3D-CISS sequence demonstrates the left AICA loop extending into the IAC (>50%). CISS = constructive interference in steady state; AICA = anterior inferior cerebellar artery; IAC = internal auditory canal

The first radiologist identified vascular loops of AICA in 108 (47.4%) of 228 sides, with 59 (54.6%) sides showing type I Chavda vascular loop (Figure 3), which was the most common type, followed by type II (Figure 4) Chavda vascular loop (Figure 2) seen in 45 sides (41.7%), and, lastly, four (3.7%) sides showing type III Chavda vascular loop (Figure 5). The second radiologist identified vascular loops of AICA in 109 (47.8%) of 228 sides. In total, 62 (56.9%) sides showed type I Chavda vascular loop, 45 (41.3%) sides presented as type II Chavda vascular loop, and two (1.8%) sides showed type III Chavda vascular loop, as depicted in Table 3.

**Table 3:** Results of the assessment of the vascular loop of AICA in bilateral CPA and IAC. The data are represented as frequency (n), percentage (%), and mean. Sample size of the study, n = 228. AICA = anterior inferior cerebellar artery; CPA = cerebellopontine angle; IAC = internal auditory canal

Variable		Radiologist 1, n	Radiologist 2, n
Presence of AICA loop - left	No	61	60
	Yes	53	54
Presence of AICA loop - right	No	59	59
	Yes	55	55
Chavda classification of AICA loop - left	Type 1	29	30
	Type 2	22	22
	Type 3	2	2
Chavda classification of AICA loop - tight	Type 1	30	32
	Type 2	23	23
	Type 3	2	0

In 119 (52.2%) of 228 sides, the absent vascular loop was agreed upon by both radiologists. Therefore, the prevalence of vascular loop in asymptomatic patients may range from 47.4% to 47.8% with a mean of 47.6%. Table 4 shows the interobserver agreement between the two radiologists. The kappa coefficient of agreement for the presence/absence of vascular loop of AICA was 0.97 (95% confidence interval = 0.94-1.00), indicating almost perfect agreement between the two radiologists. The differences in opinion between the two radiologists varied by only one grade of severity (i.e., Chavda type 1 or 2, or type 2 or 3).

**Table 4:** Interobserver agreement. The data are represented as frequency (n) and percentage (%). Sample size of the study, n = 228. CI = confidence interval

Presence of the AICA loop		Radiologist 2		Total
		Yes (n)	No (n)	
Radiologist 1	Yes (n)	106 (46.5%)	2 (0.9%)	108 (47.4%)
	No (n)	1 (0.4%)	119 (52.2%)	120 (52.6%)
Total		107 (46.9%)	121 (53.1%)	228 (100.0%)
Kappa coefficient: 0.97, 95% CI = 0.94-1.00, standard error = 0.01), percentage of agreement = 98.68%				

## Discussion

The CPA cistern houses important structures such as the trigeminal nerve, facial nerve, and vestibulocochlear nerve after they exit the brainstem. After the vestibular and cochlear roots merge, the trunk of the vestibulocochlear nerve extends anterolaterally with the VII nerve and the nervus intermedius and enters the IAC. Vestibular and cochlear roots of the VIII nerve split off at the fundus of the IAC [11].

AICA can originate from the basilar artery (98.1%) or the vertebral artery (1.9%) and as a single (92.3%) or duplicate (7.7%) artery [12]. AICA traverses closer to the abducens, facial, and vestibulocochlear nerves. In the CPA cistern, AICA lies outside the internal acoustic meatus (~19%-40%), at the meatus (33%-56%), or within the IAC (25%-27%) [13]. Vascular loops of AICA are anatomical variations that usually originate from the basilar artery (98%) or less frequently from the vertebral artery (2%) [14]. The internal auditory artery, a branch of AICA given in CPA, passes immediately into the IAC. After the internal auditory branch, AICA divides into two major branches within the CPA cistern. The lateral branch courses laterally toward the semilunar lobules and the medial branch courses toward the biventral lobule of the cerebellum [15].

The root entry/exit zone (REZ) is the transition point between the peripheral nervous system (PNS) and central nervous system (CNS), segments of a cranial nerve. Histological differences between the CNS and PNS segments are that the peripheral segment is more resilient to compression than the central segment [16]. Due to the proximity of the REZ to the IAC, the vestibulocochlear nerve (VIII nerve) has the longest CNS section. This suggests that the entire cisternal segment of the VIII nerve, extending from the brainstem to the IAC, could be especially susceptible to compression by blood vessels.

Vascular compression syndrome describes a clinical entity characterized by compression of one of the cranial nerves by a vessel. Microvascular decompression for intractable vertigo was originally performed by Janetta et al. [17]. According to one theory, at the intersection of the central glial and peripheral non-glial junctions, continuous or pulsatile compression may result in localized demyelination, reorganization, and axonal hyperactivity [18-20]. The other theory is that decreased vascular perfusion could be the outcome of diminished blood flow caused by neurovascular compression [21]. The prevalence of vascular loops affecting the VIII nerve in the REZ, where the nerve leaves the CPA without a myelin sheath, ranges from 7% to 23% [22].

Microvascular decompression is a surgical technique wherein the culprit vessel compressing the nerve root entry zone is identified and mobilized away from the nerve. Small pads of woven Teflon (pledgets) are introduced between the nerve and the vessel [23,24]. Decompression of the vestibulocochlear nerve with the opening of the IAC and transposition of the vascular loop of AICA has shown to be an effective treatment modality for the intrameatal vascular compression of the cranial nerve VIII causing tinnitus, vertigo, and hearing loss [25].

Few studies have shown a correlation between the vascular loop and audiological symptoms, albeit the majority did not show a correlation [26,27]. Mejía-Quiñones et al. observed an association between vascular loops and tinnitus, but not with sensorineural hearing loss, nystagmus, or vertigo [26]. In contrast to the non-pulsatile tinnitus group, Nowé et al. demonstrated that patients with arterial pulsatile tinnitus had a notably greater quantity of vascular loops in the IAC [27].

According to Zhang et al., about 45.5% of contralateral unaffected ears of unilateral idiopathic sudden sensorineural hearing loss (ISSNHL) patients had the AICA entering the IAC [28]. When the AICA enters the IAC (Chavda type II vascular loop) or crosses between the seventh and eighth cranial nerves (Gorrie type C), the severity and frequency of hearing impairment in ISSNHL will be affected but not the occurrence of ISSNHL [28]. According to Papadopoulou et al., a larger group of patients (70%) did not exhibit any correlation between otovestibular symptoms and vascular loops, suggesting that vascular loops of AICA are anatomical variations in a substantial majority of cases with a less common subset causing some audiovestibular symptoms [29]. Kim et al. also observed an insignificant difference in the position and neurovascular contact of the AICA loop between ears with and without ISSNHL based on the Chavda and Gorrie classification [30]. Although few studies showed some correlation between vascular loops and audiovestibular symptoms, most did not show a significant correlation.

In concordance with most studies done in symptomatic patients, our study also showed a high prevalence (47.8%) of the AICA loop, but in asymptomatic individuals. Thus, the AICA loop in the CPA cistern and IAC likely represents an anatomical variation.

The limitations of our study are the lack of correlation with a cohort of patients presenting with otovestibular symptoms and the lack of comparison between other existing classification systems for the vascular loop in the CPA cistern.

## Conclusions

The prevalence of AICA vascular loop in the CPA cistern is as high as 47.6%. This high incidence in asymptomatic patients is likely to be an anatomical variant. Therefore, just the radiological evidence of the vascular loop should not amount to pathology but should be further evaluated only to consider the AICA loop as an etiology of exclusion. Knowledge regarding the high prevalence of the AICA loop in the asymptomatic population and the lack of significant correlation between the presence of the AICA loop and otovestibular symptoms should be considered in preoperative planning for decompression procedures. Hence, it is pivotal to consider the MRI findings along with clinical features in evaluating the candidacy of symptomatic individuals for microvascular decompression to avoid unnecessary or futile interventions.
